# Phenotypic heterogeneity shapes phage resistance and cocktail efficacy in *Klebsiella pneumoniae*

**DOI:** 10.1128/spectrum.02261-25

**Published:** 2026-04-30

**Authors:** Lucas Mora-Quilis, Rafael Sanjuán, Pilar Domingo-Calap

**Affiliations:** 1 Institute for Integrative Systems Biology, University of Valencia-CSIC, Paterna, Spain; The University of Tennessee Knoxville, Knoxville, Tennessee, USA

**Keywords:** *Klebsiella pneumoniae*, phage resistance, phage cocktail, capsule reversion, phenotypic heterogeneity

## Abstract

The emergence of phage-resistant bacteria poses a significant challenge to the success of phage therapy. Although phage cocktails can delay resistance, their efficacy relies on the ability to target the full spectrum of resistant variants, which are often more diverse than those represented by clonal isolates. In this study, we investigated how phenotypic diversity within phage-resistant *Klebsiella pneumoniae* influences susceptibility to newly isolated phages, and the effectiveness of phage cocktails. We isolated phages using three cultures resistant to a capsule-dependent phage: a heterogeneous acapsular population, an acapsular mutant with a stable phenotype, and a capsule-reverted isolate that regained capsule expression upon removal of phage pressure. These phages differed in host tropism and in their capacity to delay resistance emergence when combined with the capsule-dependent phage. Phages targeting acapsular variants, particularly those isolated from the heterogeneous population, were the most effective, exhibiting strong synergy. Single-cell analyses further revealed that sustained selective pressure from the capsule-dependent phage prevents capsule reversion and maintains cocktail efficacy. Overall, our results highlight the importance of accounting for phenotypic heterogeneity when designing phage therapies, and support population-level approaches for optimizing phage cocktail composition.

**IMPORTANCE:**

Phage therapy is a promising alternative to antibiotics, but its success is often limited by the rapid emergence of phage-resistant bacteria. These resistant populations can be highly heterogeneous, comprising both stable mutants and variants with reversible, non-genetic resistance. In this study, we explore how this phenotypic diversity influences the effectiveness of phage cocktails. By isolating new phages and testing them in combination, we demonstrate that the selective pressure exerted by specific phages can prevent the reversion in transiently resistant variants, thereby sustaining treatment efficacy. Our findings highlight the need to consider not only the range of bacterial targets but also how phage pressure shapes bacterial population dynamics. This work offers a more refined strategy for designing phage cocktails with improved clinical potential.

## INTRODUCTION

Phage therapy has re-emerged in recent years as a promising alternative or complement to traditional antibiotic treatments in the fight against multi-drug resistant bacteria (1, 2). Numerous clinical cases have been reported, and the number of clinical trials involving phage treatments has grown significantly in recent years (3, 4). However, the rapid emergence of phage-resistant bacteria remains one challenge limiting the efficacy of phage therapy (5–8). To address this issue, phage cocktails, combinations of two or more phages administered simultaneously, have been proposed as a strategy to both broaden host range and suppress resistance. Bacteria possess a vast and diverse array of mechanisms to evade phage infection that include adaptive immunity (CRISPR-Cas) (9), innate defense, such as restriction-modification systems (10), programmed cell death through the abortive infection system (11), and many others (12, 13). However, one of the simplest and most effective strategies to prevent phage infection is the modification of cell surface structures that phages use for host recognition. In *Klebsiella* spp., an enterobacterium with high antibiotic resistance rates, the capsule is the primary factor determining phage infectivity (14, 15). Consequently, mutations that lead to capsule loss or modification can confer resistance to capsule-dependent phages, with the trade-off of exposing other surface structures, such as lipopolysaccharides (LPS) or outer membrane proteins (OMPs), that may serve as receptors for other phages (16–19). Importantly, capsule expression is not governed by a single gene, but is influenced by a large set of genetic and regulatory factors, leading to substantial variability in capsule quantity and architecture (20–22). Similarly, LPS and OMP can also vary among cells within the population (23, 24), further diversifying susceptibility to non-capsule-dependent phages. Modification of these bacterial structures can also result from reversible changes, such as phase variation or stochastic fluctuations in gene expression (25–28). These changes are highly dynamic and allow bacteria to rapidly switch between phenotypes, conferring a rapid adaptive strategy in changing environments, highlighting the potential adaptive advantage of transient resistance over mutation-based mechanisms. Interestingly, acapsular variants lacking the protective capsule have been shown to be less virulent, making them more susceptible to conventional antibiotics and clearance by the host immune system (29, 30). However, in immunocompromised patients or when antibiotics are no longer effective, these variants can spread rapidly and accelerate the onset of disease (31), requiring novel strategies of control. Indeed, phage cocktails composed primarily of phages targeting clonal resistant cultures have been proposed as a strategy to delay the emergence of phage resistance in *Klebsiella pneumoniae* (32–34). Nevertheless, the intrinsic heterogeneity of bacterial populations, including differential expression of the capsule components, may pose a challenge, making it difficult to capture the resistance diversity through the use of a single or a few resistant clones for phage isolation (35).

In a previous study using *K. pneumoniae* capsule type 1 as a model, we showed that resistance to the capsule-dependent phage Cap62 resulted in a heterogeneous acapsular population (36). This population was composed predominantly of reversible acapsular variants that transiently suppressed capsule expression through non-mutational mechanisms, and to a lesser extent, of stable acapsular mutants. While the mutants maintained an acapsular phenotype, the reversible variants rapidly restored capsule production once phage pressure was removed. This heterogeneity in the resistant populations poses a challenge for phage selection, as clonal isolates may not capture the full spectrum of resistance mechanisms. Therefore, here, we aimed to explore how phenotypic diversity in phage-resistant *K. pneumoniae* influences susceptibility to newly isolated phages with the potential to delay resistance emergence when used in cocktails. To this end, we isolated new phages using three Cap62-resistant cultures: the heterogeneous acapsular population, an acapsular mutant with a stable phenotype, and a capsule-reverted isolate that regained capsule expression upon removal of phage pressure. The isolated phages exhibited different host tropisms and different abilities in delaying the emergence of resistance when combined with Cap62. Phages targeting acapsular variants were more effective in delaying resistance, particularly those isolated from the heterogeneous population, which exhibited a strong synergistic effect with Cap62. Finally, single-cell experiments demonstrated that the selective pressure exerted by Cap62 is essential to prevent capsule reversion and sustain the long-term efficacy of the phage cocktail.

## RESULTS

### Isolation and characterization of phages targeting the Cap62-resistant cultures

**Fig 1:**
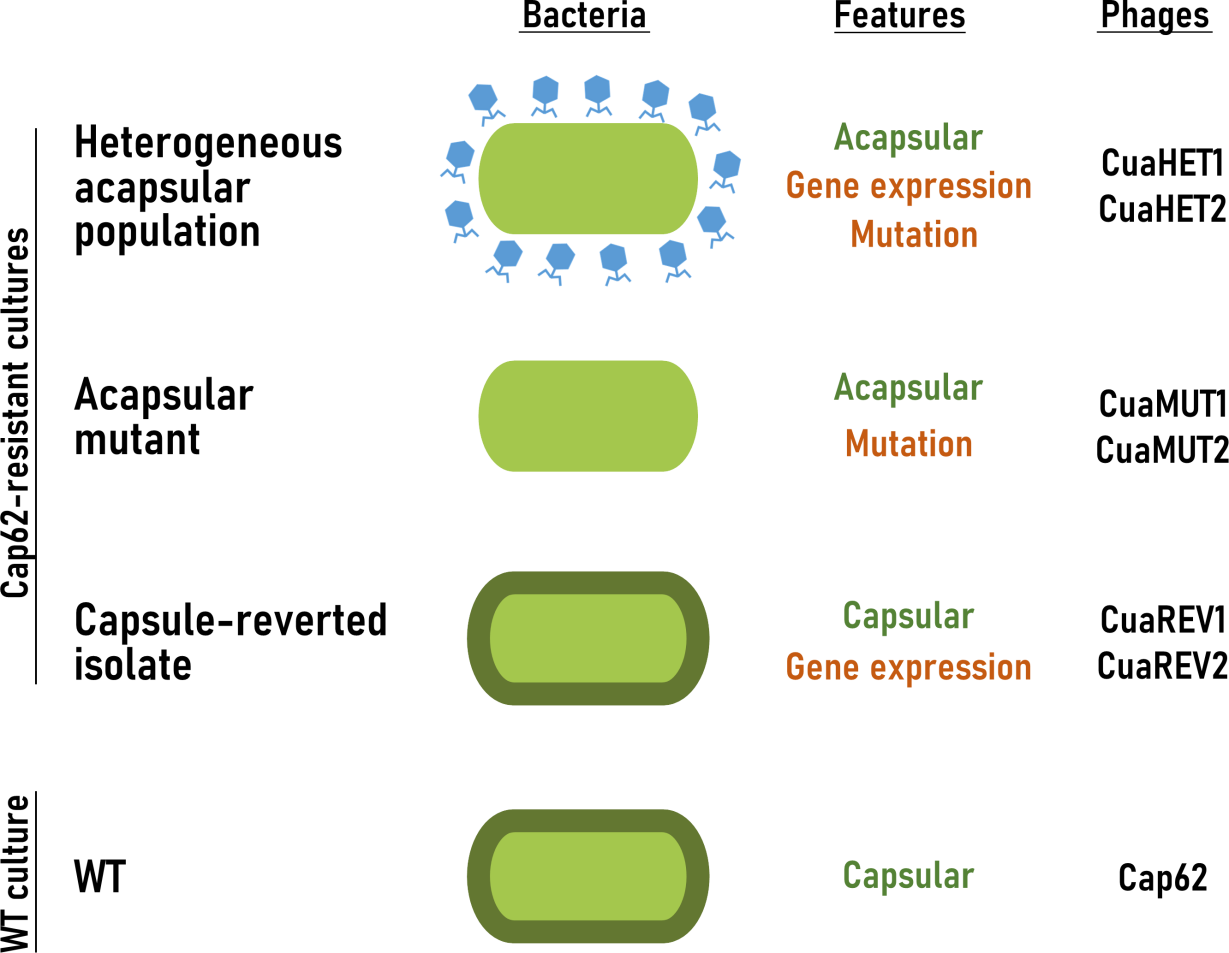
Bacterial cultures used for phage isolation. Illustrative scheme of the bacterial cultures used in this study (WT culture and Cap62-resistant cultures: heterogeneous acapsular population, acapsular mutant, and capsule-reverted isolate). Bacterial cells are depicted in green, with a dark green outline indicating the presence of a capsule in the WT and the capsule-reverted isolate. The blue phage shown with the heterogeneous acapsular population represents phage Cap62, highlighting its essential role in maintaining the acapsular phenotype. Key features indicate the presence of a capsule (green) and the resistance mechanism (orange). “Mutation” refers to stable genetic capsule loss, while “Gene expression” refers to reversible downregulation of capsule biosynthesis. Note that the heterogeneous population is primarily driven by gene expression, with acapsular mutants present at low frequency. In addition, the specific phages isolated in each strain are listed.

**Fig 2:**
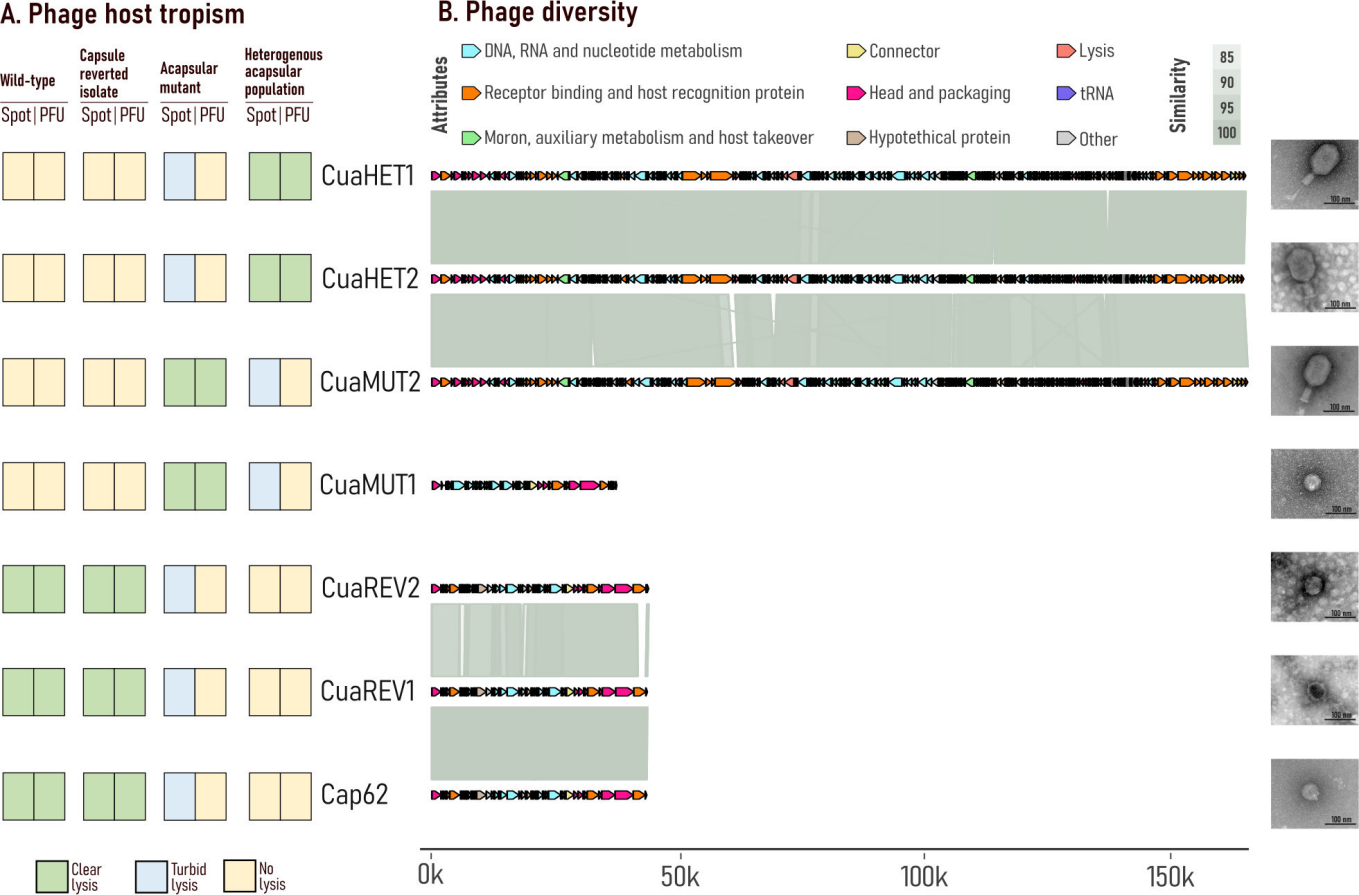
Phage host tropism and diversity. (**A**) Phage host tropism is assessed by the ability of phages to form spots or PFUs. Yellow boxes indicate no lysis, blue indicates turbid lysis, and green indicates clear lysis. (**B**) Phage diversity analysis based on the intergenomic similarity and transmission electron microscopy. Arrows represent coding sequences (CDSs) and are colored based on functional groups. The gray scale represents the percentage of genomic similarity. The genomic scale is represented in base pairs.

Cap62-resistant cultures were used as hosts for phage isolation. Phages CuaHET1 and CuaHET2 were isolated using the heterogeneous resistant population, including acapsular mutants and reversible acapsular variants. To preserve the acapsular phenotype during isolation, phage Cap62 was maintained as a selective pressure, preventing capsule restoration and enriching for acapsular cells. In addition, phages CuaMUT1 and CuaMUT2 were isolated from the acapsular mutant, and phages CuaREV1 and CuaREV2 from the capsule-reverted isolate (Fig. 1). The ability of these phages to infect the Cap62-resistant cultures was evaluated. Similar to phage Cap62, phages CuaREV1 and CuaREV2 were able to form spots and plaque-forming units (PFU) on both the capsular WT strain and the capsule-reverted isolate. In contrast, phages CuaMUT1, CuaMUT2, CuaHET1, and CuaHET2 were only able to infect and lyse acapsular-resistant cultures. Notably, these phages produced clear spots and individual PFUs, confirming productive infection, only in the specific culture from which they were isolated (Fig. 2A).

Full-genome sequencing revealed that phages CuaHET1, CuaHET2, and CuaMUT2 belonged to the *Jiaodovirus* genus (91.8%–97.4% intergenomic similarity). Phage CuaMUT1 belonged to the *Teetrevirus* genus. Finally, phages CuaREV1 and CuaREV2 belonged to the *Drulisvirus* genus, showing a high intergenomic similarity (85.1%–99.9%) with Cap62 (Fig. 2B; Fig. S1A). A virulent lifestyle was predicted for all phages (ranging from 96.2% to 100%; Fig. S1B). Depolymerase-related genes were just found in those phages belonging to the genus *Drulisvirus* and in phage CuaMUT1 (Fig. S1C). To further investigate the genetic determinants of host specificity among the closely related *Jiaodoviruses*, their structural proteins were compared. While phages CuaHET1 and CuaHET2 encode a full-length tail fiber protein and a putative adhesin (corresponding to CDS 0087 and CDS 0088 in CuaHET1), phage CuaMUT2 lacks the adhesin gene and presents a truncation in the C-terminal receptor-binding domain of the tail fiber. These structural variations in the receptor-binding modules provide a potential explanation for the distinct host tropism observed between CuaMUT2 and CuaHET-like phages.

### Evaluation of phage cocktails to counteract phage resistance

**Fig 3:**
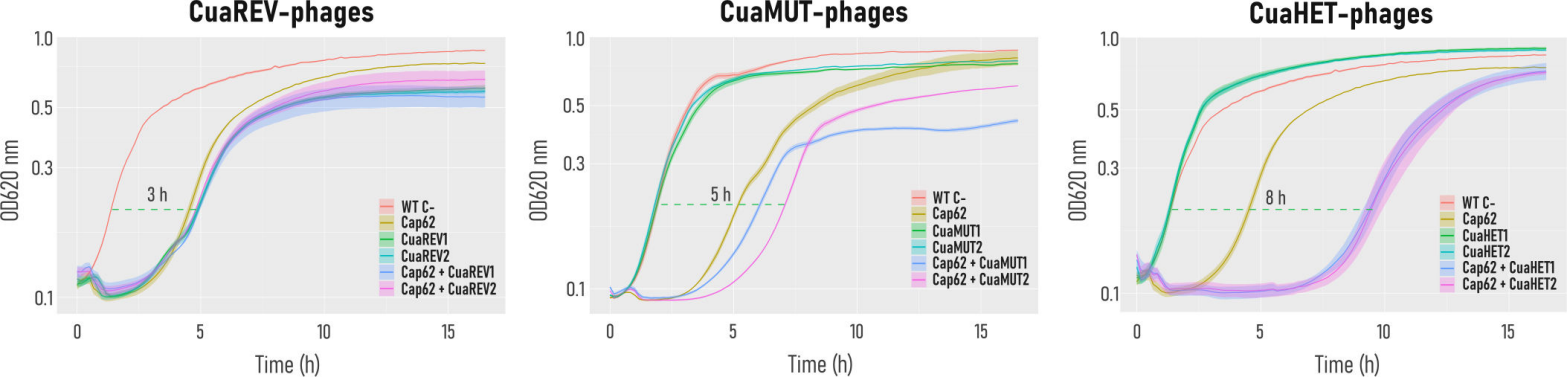
Efficacy of phage cocktails in delaying resistance emergence. Bacterial growth curves were measured by optical density at 620 nm over 16 h. WT C− represents bacterial growth without phage. Other conditions represent bacterial growth with either a single phage or a cocktail of two phages. The shaded areas denote the standard error of the mean for three replicates.

To test the ability of these newly isolated phages to delay the emergence of resistant bacteria, the infectivity of phage cocktails combining each isolated phage individually with Cap62 was evaluated against the WT culture in liquid media. The cocktails composed of CuaREV1 or CuaREV2 with Cap62 showed no delay in resistance, resulting in the same effect as that of Cap62 in monoinfection (one-way ANOVA, followed by Dunnett’s test, *P* > 0.05). Interestingly, a delay in the emergence of resistance was observed only when Cap62 was combined with phages capable of infecting acapsular cultures. While the cocktail containing CuaMUT1 showed only a trend toward delaying resistance (*P* = 0.09), the combination with CuaMUT2 significantly delayed resistance (*P* < 0.01). However, the greatest effect was shown by the combination of CuaHET1 or CuaHET2 with Cap62 (*P* < 0.001), where resistance was delayed by more than 8 h relative to the uninfected culture (Fig. 3).

### The delay in resistance is due to the complementary cell tropism by phages

To better understand the synergistic effect in delaying resistance, we focused on the phage cocktail encompassing Cap62 and CuaHET1, which proved to be one of the most effective combinations. To achieve this, the WT culture was passed through a flow cytometer, and individual cells were dispensed into wells containing different treatments: lysogenic broth (LB) medium, phage Cap62, phage CuaHET1, or a combination of both (phage cocktail). In the LB control wells, all cells were expected to grow, as no phage was present. In contrast, growth in wells containing phages was expected only from resistant cells.

**Fig 4:**
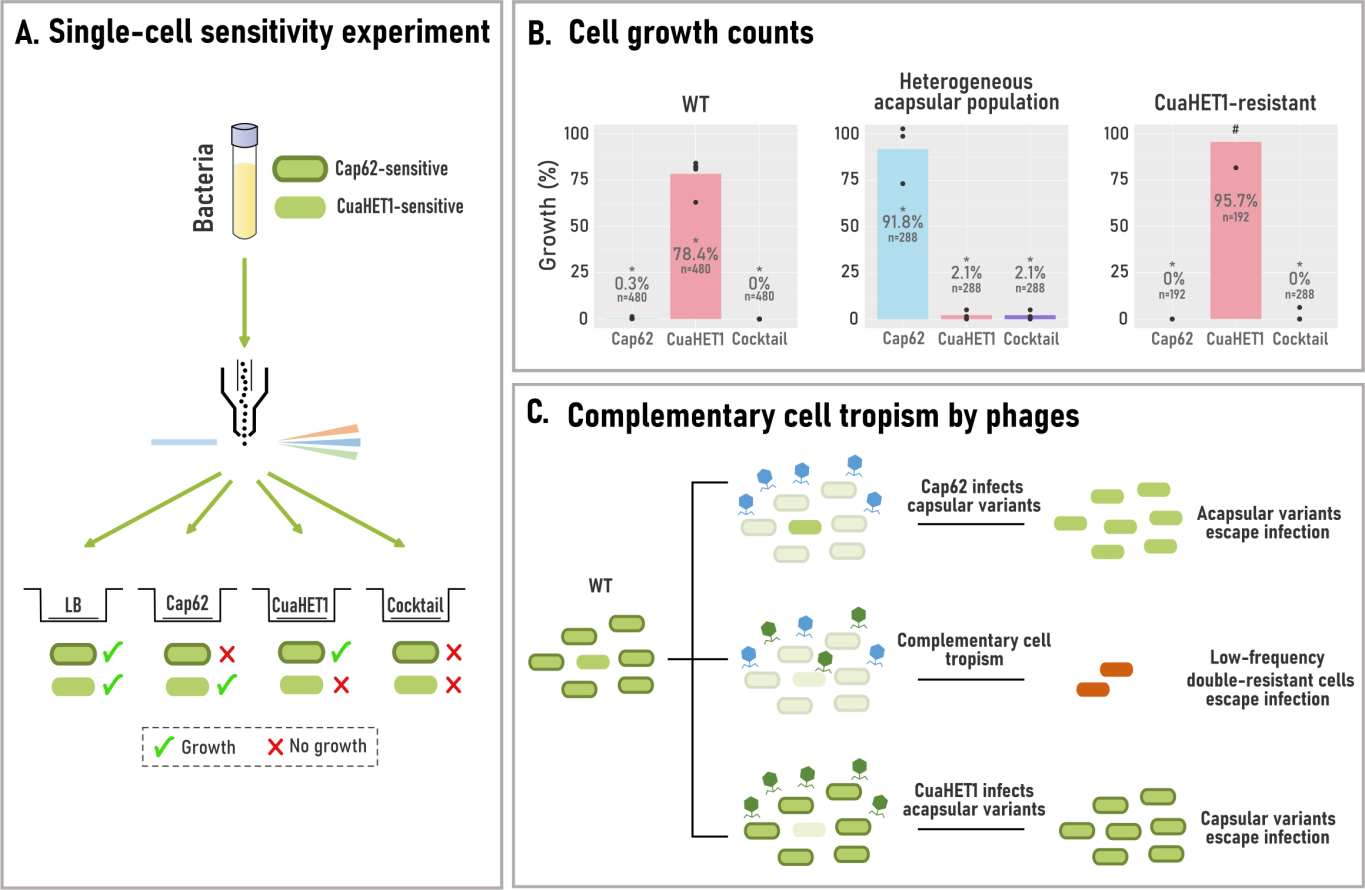
Single-cell sensitivity to phages. (**A**) Schematic representation of the single-cell sensitivity experiment. Cells from liquid cultures were sorted into individual wells containing different treatments (LB, Cap62, CuaHET1, or phage cocktail). Bacterial cells are shown in green, with a dark green outline indicating the presence of a capsule. Capsulated cells are sensitive to Cap62, while acapsular cells are sensitive to CuaHET1. A green tick indicates bacterial growth, while a red cross indicates no growth. (**B**) Percentage of wells with bacterial growth for the WT, heterogeneous acapsular, and CuaHET1-resistant populations, relative to the LB condition (100%). The mean of independent replicates is shown (*N* = 2–5, individual points), indicating the total number of analyzed cells (*n*). 0% indicates growth below the limit of detection (<0.5%), and the hash symbol (#) denotes an off-scale value (110.45%). *P < 0.05, Fisher’s exact test on pooled data, relative to the LB condition. (**C**) Schematic representation of the synergistic effect in delaying resistance emergence, driven by the complementary cell tropism of phages Cap62 (blue) and CuaHET1 (green).

After 48 h of incubation, we counted the number of wells in which bacteria had survived and exhibited growth. This allowed us to estimate the proportion of cells in each culture that were sensitive to phage Cap62 and CuaHET1 (Fig. 4A). The WT culture was predominantly composed of cells sensitive to Cap62, while 78.4% of the population was resistant to the CuaHET1 phage (Fig. 4B). This confirmed that the WT was highly susceptible to Cap62, with Cap62-resistant variants present at levels below the detection threshold (<0.2%). Notably, 21.6% of the WT population remained sensitive to CuaHET1. Finally, no growth was observed in wells treated with the phage cocktail, further indicating that cells resistant to both phages are present at undetectably low frequencies.

To assess whether resistance to Cap62 affects single-cell phage sensitivity, this assay was repeated using the heterogeneous Cap62-resistant population (Fig. 4B). In this culture, the proportion of Cap62-resistant cells increased markedly to 91.8%, accompanied by a corresponding rise in sensitivity to CuaHET1. This indicated that the acapsular phenotype promoted CuaHET1 infection. Conversely, analysis of a CuaHET1-resistant culture revealed a higher proportion of CuaHET1-resistant cells compared to the WT, while sensitivity to Cap62 was maintained. This reciprocal sensitivity likely underlies the delayed emergence of resistance observed when both phages were applied in combination (Fig. 4C).

### Cap62 selective pressure prevents capsule reversion and sustains cocktail synergy

**Fig 5:**
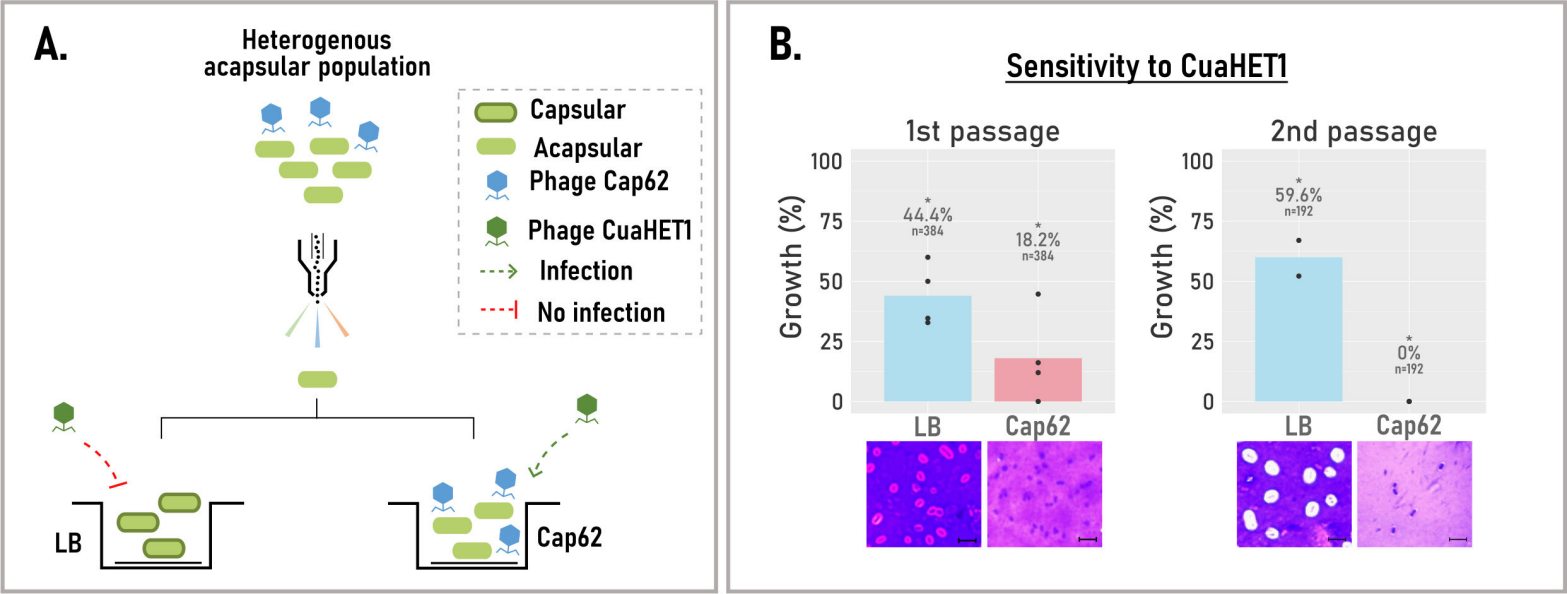
Influence of phage Cap62 on capsule dynamics and susceptibility to CuaHET1. (**A**) Schematic representation of the tested hypothesis. Cells from the heterogeneous acapsular population grown in LB restore capsule and become resistant to phage CuaHET1 (red dotted line), whereas those grown in the presence of Cap62 remain acapsular and susceptible to phage CuaHET1 (green dotted line). (**B**) Sensitivity of single-cell-derived acapsular cultures grown in LB (blue) or in the presence of Cap62 (salmon) to phage CuaHET1. Data is shown as the percentage (%) of wells with bacterial growth over two consecutive single-cell passages. Bars represent the mean of independent biological replicates (*N* = 2–4, individual points), indicating the total number of analyzed cells (*n*). 0% indicates growth below the limit of detection (<0.5%). *P < 0.05, Fisher’s exact test on pooled data, relative to the LB condition. Microscopy images show that cells grown in LB restore capsule production, while cells grown in Cap62 retain the acapsular phenotype. The scale bars represent 5 µm.

The synergistic effect of the phage cocktail was driven by the differential lytic activity of both phages, Cap62 targeting capsular variants, and phage CuaHET1 acapsular ones. However, as previously shown, the resistant culture represented a heterogeneous population of acapsular variants. In the absence of Cap62-mediated selective pressure, some of these variants reverted to capsule production (e.g., the capsule-reverted isolate), while others remained acapsular (e.g., the acapsular mutant). This capacity for capsule reversion could undermine the long-term efficacy of the phage cocktail. To determine whether the presence of Cap62 was required to maintain CuaHET1 susceptibility, we isolated individual cells from the heterogeneous acapsular resistant population and allowed them to grow in wells containing either LB or phage Cap62 (Fig. 5A). Following growth, cultures were subjected to single-cell sorting to assess their sensitivity to CuaHET1 and examined under the microscope to evaluate the presence of capsule (Fig. 5B). The results indicated that acapsular cells grown in LB restored capsule production, leading to increased resistance to CuaHET1 (44.4% growth) compared to the original resistant population. In contrast, cells grown in the presence of Cap62 maintained the acapsular state and exhibited higher susceptibility to CuaHET1 (18.2% growth). Upon a subsequent passage, this trend was even more pronounced: cells grown in LB exhibited further increased resistance to CuaHET1 (59.6% growth), whereas those maintained under Cap62 pressure remained fully sensitive (<0.5% growth). These findings suggest that Cap62 is essential for preserving the acapsular phenotype, thereby sustaining CuaHET1 efficacy within the cocktail.

## DISCUSSION

The emergence of phage-resistant bacteria is a major challenge for phage therapy (5). While phage cocktails have shown efficacy in delaying resistance, their effectiveness depends on the ability to target the full spectrum of resistant variants (37). Most studies isolated and characterized resistance based on clonal cultures (33, 38, 39). However, phage-resistant populations often display substantial genetic and phenotypic diversity, which cannot be only represented by a single clone (40). In this study, we demonstrated that distinct subpopulations within phage-resistant *K. pneumoniae* cultures displayed differential susceptibility to phages, highlighting how population heterogeneity can shape phage-host interactions and influence cocktail efficacy.

Previous studies have shown that capsule-deficient mutants facilitate the isolation of phages that specifically target acapsular variants, while capsulated strains typically yield capsule-dependent phages (14, 19, 41). In some cases, multiple acapsular mutants were isolated to cover the full resistance spectrum (33, 38, 39). However, this approach is not always feasible, especially when the resistant population includes transient acapsular variants (36). In such cases, applying a selective pressure, such as a capsule-targeting phage, can help to stabilize the acapsular state and prevent capsule reversion, thereby enhancing the isolation of phages that target these unstable phenotypes. Our results demonstrate that applying this selective pressure not only stabilized the bacterial population but also directly shaped the host specificity of the isolated phages, favoring the selection of those capable of infecting the transient acapsular variants. Phages capable of targeting a broader range of resistant variants are generally more effective at delaying resistance emergence (19, 32, 33). This effect is often attributed to complementary host tropism, where each phage targets a distinct subset of the bacterial population (37, 42). Therefore, combinations of phages with overlapping host ranges, such as Cap62 and CuaREV-like phages, may be less effective due to functional redundancy. In contrast, combinations that include phages showing lytic activity against distinct bacterial subpopulations, such as capsulated and acapsulated variants, are more likely to exert broad selective pressure and reduce the chances of resistant escape.

Isolating phages from heterogeneous resistant populations may increase the chance of selecting phages with broader host ranges, whereas relying on clonal cultures like acapsular mutants may limit this potential (43). However, this study suggests that the superior efficacy of CuaHET-like phages compared to CuaMUT-like phages is likely not driven by a broader host range, as CuaHET-like phages were unable to effectively infect the acapsular mutant culture. An alternative explanation is the presence of a dominant subpopulation within the heterogeneous resistant culture. Phages able to target this subpopulation may exert stronger selective pressure (44), resulting in enhanced suppression of resistance compared to phages targeting less abundant subpopulations. Although capsule biosynthesis in *K. pneumoniae* is primarily governed by the K-locus; additional genes can modulate capsule quantity and architecture, potentially affecting access to alternative receptors, such as LPS or OMPs (21). Moreover, previous literature suggested that LPS and OMP expression may also vary across cells within the population (23, 24). Although specific receptor analysis was beyond the scope of this study, such surface heterogeneity could explain why the acapsular phenotypes of a mutant clone and a reversible acapsular variant displayed different susceptibility to non-capsule-dependent phages, as observed for CuaHET-like and CuaMUT-like phages. Consistent with this, our genomic analysis revealed significant structural divergence in the receptor-binding modules of these phages, specifically the loss of a putative adhesin and the truncation of the tail fiber receptor-binding domain in CuaMUT2. These specific adaptations likely reflect the distinct surface architecture of the host populations from which they were isolated.

Our previous findings demonstrated that, although acapsular mutants are present in the resistant population, mutation-driven acapsularity is not the predominant resistance mechanism (36). Instead, resistance mostly arises from transient acapsular states. While our previous transcriptomic analysis revealed the down-regulation of capsule biosynthesis genes (36), the current single-cell experiments functionally confirmed the rapid restoration of capsule expression. This reversible phenotypic switch is consistent with mechanisms described in the literature, such as phase variation or stochastic fluctuations in gene expression, which facilitate adaptation to challenging environments (45). While spontaneous mutations in bacteria occur at a relatively low frequency, around 10⁻¹⁰ mutations per nucleotide per generation (46, 47), phase variation can occur at rates up to three orders of magnitude higher (25). Stochastic gene expression changes are even more frequent, generating phenotypic variability on much shorter timescales (27, 28, 48, 49). Although the precise molecular mechanism remains to be elucidated, the rapid reversion rates observed in our single-cell assays align with these high-frequency switching models. Consequently, the superior ability of CuaHET-like phages to delay resistance may stem from their capacity to lyse non-mutational acapsular variants, which likely emerge more frequently than acapsular mutants in the population.

Single-cell experiments underscored the dynamic interplay between phage pressure and bacterial phenotype. The efficacy of CuaHET-like phages is tightly linked to the selective pressure exerted by a capsule-targeting phage, which is necessary to maintain the acapsular state. Once this pressure is removed, capsule expression can be rapidly restored, reducing phage susceptibility. These observations support the idea that simultaneous administration of complementary phages may be more effective than sequential treatments in capsular bacteria, as previously suggested (50), as it maintains continuous selective pressure and prevents phenotypic reversion. While acapsular variants are typically cleared by the host immune system (29), such clearance may be insufficient in immunocompromised patients. In this clinical scenario, the persistence of acapsular variants could facilitate capsule restoration and subsequent treatment relapse once the primary phage pressure is removed. Consequently, incorporating phages that target these transient variants may be critical not only to broaden host range but specifically to block this escape trajectory.

In conclusion, our findings highlight the importance of considering phenotypic diversity within bacterial populations when designing phage therapies. The effectiveness of phage cocktails depends not only on combining phages targeting distinct receptors but also on understanding how selective pressures shape population phenotype and influence phage susceptibility. These results underscore the limitations of conventional phage selection strategies and support population-level approaches to improve the efficacy of phage therapy.

## MATERIALS AND METHODS

### Bacterial strains and phage Cap62

The reference *K. pneumoniae* strain used in this study corresponds to the capsular type 1 strain (referred to as WT), obtained from the Statens Serum Institut (Copenhagen, Denmark). Phage Cap62 and the three derived phage-resistant cultures were described previously (36). These cultures include the heterogeneous acapsular population composed primarily of reversible acapsular variants, along with a minority of stable acapsular mutants; an acapsular mutant with a stable phenotype; and a capsule-reverted isolate that harbored no detectable mutations and restored capsule production through changes in gene expression. All bacterial cultures were grown in LB supplemented with 3.78 mM CaCl_2_ to promote phage adsorption at 37°C with shaking.

### Isolation and purification of phages

Wastewater samples from the metropolitan area of Valencia (Spain) were centrifuged at 4°C, 4,000 × *g* for 10 min to pellet dust and large particles. The supernatant was filtered through 0.22 µm filters, and 800 µL was mixed with 200 µL of a Cap62-resistant culture in the stationary phase. Specifically, for the isolation of phages targeting the heterogeneous population, phage Cap62 was maintained in the bacterial suspension to prevent capsule restoration. The mixture was incubated at room temperature for 15 min, and then mixed with 3.5 mL of semi-solid LB agar and poured onto LB agar plates. After an overnight incubation at 37°C, lytic plaques were selected for plaque-to-plaque purification with a minimum of three passages. For each passage, plaques were resuspended with LB, centrifuged to pellet bacteria, and the supernatants were diluted and plated for the next passage. The supernatant from the final purification passage was amplified in LB + CaCl_2_ using 10^6^–10^7^ colony-forming units (CFU)/mL and an initial phage concentration ranging between 10^6^ and 10^7^ PFU/mL. After approximately 3 h of incubation at 37°C with shaking, samples were centrifuged at 18,000 × *g* for 3 min to pellet bacteria, and the supernatant was filtered through 0.22 µm filters. Finally, samples were aliquoted and stored at −70°C.

### Phage infectivity via spot assay and plaque-forming unit analysis

To assess the efficiency of phages in infecting the phage-resistant cultures, spot and plaque assays were done to test the ability of phages to form PFUs. For spot-tests, 200 µL of a stationary-phase culture containing approximately 10^9^ CFU was mixed with 3.5 mL of semi-solid agar LB and plated onto an LB agar plate. After drying, 1 µL of phage was spotted at a concentration of approximately 10^8^ PFU/mL, followed by an overnight (ON) incubation at 37°C. Additionally, 10-fold serial dilutions were routinely spotted. To evaluate the ability of phages to form PFU in the resistant cultures, serial dilutions of the phage were prepared, and 10 µL of each dilution was plated with bacteria in semi-solid LB agar, as previously described. After ON incubation at 37°C, the presence of PFUs on the agar plates was assessed. Each experiment was evaluated in at least three independent experiments, and the results were scored as clear lysis, turbid lysis, or no lysis.

### Bacterial growth curve assay to evaluate phage cocktail efficacy

To test the ability of cocktails to delay the emergence of resistant bacteria, the newly isolated phages were individually combined with Cap62 in liquid infections. Approximately 5 × 10^6^ CFU of the WT culture was mixed with phages at a total multiplicity of infection (MOI) of 0.5 in a final volume of 150 µL. This MOI was selected to allow for multiple rounds of infection while monitoring resistance emergence. Crucially, in the combined treatments, the amount of each phage was half that used in the mono-infection to maintain the same total phage concentration, ensuring that cocktail efficacy resulted from phage complementarity rather than a different viral input. To monitor phage-induced lysis and the growth of resistant bacteria, we measured OD_620_ nm over 16 h with continuous shaking at 37°C, using 96-well plates and a plate reader (Multiskan FC). Experiments were performed in triplicate. We determined the delay in resistance emergence by calculating the difference in time required to reach the maximum growth rate (maximum slope) between the infected and uninfected cultures. The slope was calculated as $m=\frac{{OD}_{n+1}-{OD}_{n}}{T_{n+1}-T_{n}}$, where OD represents the OD_620_ nm, and T is the time.

### Capsule staining

To determine the presence of the capsule in bacterial cultures, contrast staining with crystal violet and nigrosine was performed as described in reference 51. Briefly, liquid cultures with a minimum recommended concentration of 5 × 10^9^ CFU/mL were fixed for 20 min with 2.5% formaldehyde in the presence of 100 mM lysine. The fixative agent was removed by centrifugation and washed with 1× phosphate-buffered saline (PBS). A total of 25–40 µL of the fixed culture was mixed with a small drop of nigrosine 10% in a glass slide and smeared along the slide until air dried. One percent crystal violet was gently poured to cover the slide and incubated for 5 min at room temperature. The preparation was carefully rinsed with distilled water, air dried, and examined under a light microscope.

### Single-cell sensitivity to phages

The WT culture was grown in LB medium alone and in the presence of phage (10^8^ PFU/mL) to generate the phage-resistant cultures. To remove the broth culture and partially eliminate the surrounding phage, cultures were washed five times with 1× PBS. These were then resuspended in PBS to a final concentration of 10^5^ CFU/mL. The cultures were subsequently passed through a cytometer (BD FACSAria Fusion) under biosafety level-2 conditions to isolate individual cells into 96-well plates containing different treatments: LB, phage Cap62, phage CuaHET1, or a cocktail of both phages. Phage concentrations were maintained extremely high (10^8^–10^9^ PFU/mL) to ensure that only resistant bacteria could grow. Plates were incubated for 48 h at 37°C, and the number of wells in which bacteria grew was counted. Experiments were performed in independent biological replicates (*N* = 2–5), comprising a total of 192 to 480 single cells analyzed per condition. Consequently, the limit of detection for this assay ranged from approximately 0.2% to 0.5%, depending on the total number of cells analyzed. To calculate percentages, the number of wells with bacterial growth in LB medium was considered as the maximum (100%). The proportion of wells with bacterial growth in the presence of phage was then normalized to this maximum growth to determine the relative resistance of the bacteria to the phages. Statistical differences were analyzed using Fisher’s exact test on the pooled data set.

To analyze the effect of phage selective pressure on the maintenance of acapsularity, we performed a first passage, isolating single cells from the heterogeneous acapsular population into wells containing LB or Cap62 (10^8^–10^9^ PFU/mL). After 48 h incubation at 37°C, cultures were subjected to a second passage via single-cell sorting, in which individual cells were again isolated in LB or Cap62. Both passages were subjected to cell sorting, and individual cells were exposed to LB and Cap62 (10^8^–10^9^ PFU/mL) treatments. Plates were incubated, and the number of wells with bacterial growth was counted as previously described.

### Transmission electron microscopy of phages

Phage lysates were filtered through 0.22 µm and concentrated by centrifugation at 80,000 × *g*, 4°C, 2 h to obtain a minimum titer of 10^10^ PFU/mL. The pellet was resuspended in SM buffer, and a small volume was placed on a carbon-coated Formvar supported by a 300 mesh copper grid. Preparations were air-dried for 30 min, and any excess of liquid was removed with filter paper. The samples were then negatively stained with 2% phosphotungstic acid and observed using a Jeol JEM-1010 electron microscope.

### Phage sequencing and genomic analysis

Phage lysates were filtered through 0.22 µm and concentrated using the Concentrating Pipette Select (Innovaprep) to reach a minimum titer of 10^10^ PFU/mL. A total of 180 µL of the concentrated lysate was used to extract DNA from viral capsids using an automated protocol with the Maxwell RSC Instrument (Promega). DNA libraries were generated using the Illumina Nextera XT DNA kit (2 × 150 bp or 2 × 250 bp paired-end reads), and the Illumina MiSeq platform was used to generate the sequencing reads with the MiSeq Reagent Kit v2. Read quality was checked using FastQC (52), confirming mean quality scores > Q30, and genomes were assembled *de novo* using SPAdes v3.15.4 (53) in a single contig. To validate the assembly, reads were mapped back to the assembled genomes using BWA-MEM (54). Sequencing depth exceeded 250× for all isolates (range: 280× to 7,000×) with 100% genome coverage. Phage genomes were annotated using Pharokka (55). Genes were classified into functional groups assigned by PHROGs, and a comparative plot of the genomes was made using gggnomes (56). BACPHLIP (57) was used to predict the lifestyle of phages. And VIRIDIC (58) was used to estimate the intergenomic similarity between phages. Protein sequences were compared using BLASTp (59). To predict depolymerase activity in phage coding sequences (CDS), three tools, DepoScope (60), PhageDPO (61), and DePP (62), were used. Proteins were considered depolymerases only if all three predictors returned a score ≥0.90.

## Data Availability

Complete genome sequences of the phages used in this study are available in the NCBI GenBank under the following accession numbers: Cap62 (PQ741857), CuaREV1 (PX060902), CuaREV2 (PX060903), CuaMUT1 (PX060898), CuaMUT2 (PX060899), CuaHET1 (PX060900), CuaHET2 (PX060901). The bacterial strains used in this study were previously described (36).

## SUPPLEMENTAL MATERIAL

Fig. S1 spectrum.02261-25-s0001.pdf Genomic characterization of isolated phages.

ASM does not own the copyrights to Supplemental Material that may be linked to, or accessed through, an article. The authors have granted ASM a non-exclusive, world-wide license to publish the Supplemental Material files. Please contact the corresponding author directly for reuse.

## References

[1] Strathdee SA, Hatfull GF, Mutalik VK, Schooley RT. 2023. Phage therapy: from biological mechanisms to future directions. Cell 186:17–31. doi:10.1016/j.cell.2022.11.017 36608652 PMC9827498

[2] Skurnik M, Alkalay-Oren S, Boon M, Clokie M, Sicheritz-Pontén T, Dąbrowska K, Hatfull GF, Hazan R, Jalasvuori M, Kiljunen S, Lavigne R, Malik DJ, Nir-Paz R, Pirnay J-P. 2025. Phage therapy. Nat Rev Methods Primers 5:9. doi:10.1038/s43586-024-00377-5

[3] Pirnay J-P, Djebara S, Steurs G, Griselain J, Cochez C, De Soir S, Glonti T, Spiessens A, Vanden Berghe E, Green S, et al.. 2024. Personalized bacteriophage therapy outcomes for 100 consecutive cases: a multicentre, multinational, retrospective observational study. Nat Microbiol 9:1434–1453. doi:10.1038/s41564-024-01705-x 38834776 PMC11153159

[4] Uchechukwu CF, Shonekan A. 2024. Current status of clinical trials for phage therapy. J Med Microbiol 73:001895. doi:10.1099/jmm.0.001895 39320361 PMC11423923

[5] Green SI, Clark JR, Santos HH, Weesner KE, Salazar KC, Aslam S, Campbell JW, Doernberg SB, Blodget E, Morris MI, Suh GA, Obeid K, Silveira FP, Filippov AA, Whiteson KL, Trautner BW, Terwilliger AL, Maresso A. 2023. A retrospective, observational study of 12 cases of expanded-access customized phage therapy: production, characteristics, and clinical outcomes. Clin Infect Dis 77:1079–1091. doi:10.1093/cid/ciad335 37279523 PMC10573729

[6] Blasco L, López-Hernández I, Rodríguez-Fernández M, Pérez-Florido J, Casimiro-Soriguer CS, Djebara S, Merabishvili M, Pirnay J-P, Rodríguez-Baño J, Tomás M, López Cortés LE. 2023. Case report: analysis of phage therapy failure in a patient with a Pseudomonas aeruginosa prosthetic vascular graft infection. Front Med 10. doi:10.3389/fmed.2023.1199657 PMC10235614 37275366

[7] Qin J, Wu N, Bao J, Shi X, Ou H, Ye S, Zhao W, Wei Z, Cai J, Li L, Guo M, Weng J, Lu H, Tan D, Zhang J, Huang Q, Zhu Z, Shi Y, Hu C, Guo X, Zhu T. 2020. Heterogeneous Klebsiella pneumoniae co-infections complicate personalized bacteriophage therapy. Front Cell Infect Microbiol 10:608402. doi:10.3389/fcimb.2020.608402 33569355 PMC7868542

[8] Oechslin F. 2018. Resistance development to bacteriophages occurring during bacteriophage therapy. Viruses 10:351. doi:10.3390/v10070351 29966329 PMC6070868

[9] Rath D, Amlinger L, Rath A, Lundgren M. 2015. The CRISPR-Cas immune system: biology, mechanisms and applications. Biochimie 117:119–128. doi:10.1016/j.biochi.2015.03.025 25868999

[10] Bickle TA, Krüger DH. 1993. Biology of DNA restriction. Microbiol Rev 57:434–450. doi:10.1128/mr.57.2.434-450.1993 8336674 PMC372918

[11] Lopatina A, Tal N, Sorek R. 2020. Abortive infection: bacterial suicide as an antiviral immune strategy. Annu Rev Virol 7:371–384. doi:10.1146/annurev-virology-011620-040628 32559405

[12] Georjon H, Bernheim A. 2023. The highly diverse antiphage defence systems of bacteria. Nat Rev Microbiol 21:686–700. doi:10.1038/s41579-023-00934-x 37460672

[13] Beavogui A, Lacroix A, Wiart N, Poulain J, Delmont TO, Paoli L, Wincker P, Oliveira PH. 2024. The defensome of complex bacterial communities. Nat Commun 15:2146. doi:10.1038/s41467-024-46489-0 38459056 PMC10924106

[14] Beamud B, García-González N, Gómez-Ortega M, González-Candelas F, Domingo-Calap P, Sanjuan R. 2023. Genetic determinants of host tropism in Klebsiella phages. Cell Rep 42:112048. doi:10.1016/j.celrep.2023.112048 36753420 PMC9989827

[15] Haudiquet M, Le Bris J, Nucci A, Bonnin RA, Domingo-Calap P, Rocha EPC, Rendueles O. 2024. Capsules and their traits shape phage susceptibility and plasmid conjugation efficiency. Nat Commun 15:2032. doi:10.1038/s41467-024-46147-5 38448399 PMC10918111

[16] Hesse S, Rajaure M, Wall E, Johnson J, Bliskovsky V, Gottesman S, Adhya S. 2020. Phage resistance in multidrug-resistant Klebsiella pneumoniae ST258 evolves via diverse mutations that culminate in impaired adsorption. mBio 11:e02530-19. doi:10.1128/mBio.02530-19 31992617 PMC6989104

[17] Kortright KE, Chan BK, Turner PE. 2020. High-throughput discovery of phage receptors using transposon insertion sequencing of bacteria. Proc Natl Acad Sci USA 117:18670–18679. doi:10.1073/pnas.2001888117 32675236 PMC7414163

[18] Gao D, Ji H, Wang L, Li X, Hu D, Zhao J, Wang S, Tao P, Li X, Qian P. 2022. Fitness trade-offs in phage cocktail-resistant Salmonella enterica serovar enteritidis results in increased antibiotic susceptibility and reduced virulence. Microbiol Spectr 10:e0291422. doi:10.1128/spectrum.02914-22 36165776 PMC9603643

[19] Lourenço M, Osbelt L, Passet V, Gravey F, Megrian D, Strowig T, Rodrigues C, Brisse S. 2023. Phages against noncapsulated Klebsiella pneumoniae: broader host range, slower resistance. Microbiol Spectr 11:e0481222. doi:10.1128/spectrum.04812-22 37338376 PMC10433977

[20] Wyres KL, Wick RR, Gorrie C, Jenney A, Follador R, Thomson NR, Holt KE. 2016. Identification of Klebsiella capsule synthesis loci from whole genome data. Microb Genom 2:e000102. doi:10.1099/mgen.0.000102 28348840 PMC5359410

[21] Dorman MJ, Feltwell T, Goulding DA, Parkhill J, Short FL. 2018. The capsule regulatory network of Klebsiella pneumoniae defined by density-TraDISort. mBio 9:e01863-18. doi:10.1128/mBio.01863-18 30459193 PMC6247091

[22] Nucci A, Le Bris J, Diaz-Diaz S, Torres-Elizalde L, Rocha EPC, Rendueles O. 2025. Phenotypic heterogeneity of capsule production across opportunistic pathogens. mBio 16:e0180725. doi:10.1128/mbio.01807-25 40905702 PMC12505892

[23] Simpson BW, Trent MS. 2019. Pushing the envelope: LPS modifications and their consequences. Nat Rev Microbiol 17:403–416. doi:10.1038/s41579-019-0201-x 31142822 PMC6913091

[24] Cao X, Cao C, Chen Z, Li J, Yao Z, Zheng Y, Wu J, Li Z, Hu Y, Hao G, Zhu G, Köster W, White AP, Wang Y. 2025. Genome-wide investigation of outer membrane protein families under mosaic evolution in Escherichia coli. Appl Environ Microbiol 91:e0055725. doi:10.1128/aem.00557-25 40444982 PMC12175518

[25] van der Woude MW, Bäumler AJ. 2004. Phase and Antigenic Variation in Bacteria. Clin Microbiol Rev 17:581–611. doi:10.1128/CMR.17.3.581-611.2004 15258095 PMC452554

[26] Elowitz MB, Levine AJ, Siggia ED, Swain PS. 2002. Stochastic gene expression in a single cell. Science 297:1183–1186. doi:10.1126/science.1070919 12183631

[27] Chapman-McQuiston E, Wu XL. 2008. Stochastic receptor expression allows sensitive bacteria to evade phage attack. Part I: experiments. Biophys J 94:4525–4536. doi:10.1529/biophysj.107.120212 18310238 PMC2480656

[28] Chapman-McQuiston E, Wu XL. 2008. Stochastic receptor expression allows sensitive bacteria to evade phage attack. Part II: theoretical analyses. Biophys J 94:4537–4548. doi:10.1529/biophysj.107.121723 18310241 PMC2480678

[29] Merino S, Tomás JM. 2015. Bacterial capsules and evasion of immune responses, p 1–10. *In* eLS. John Wiley & Sons, Ltd.

[30] Henke MT, Brown EM, Cassilly CD, Vlamakis H, Xavier RJ, Clardy J. 2021. Capsular polysaccharide correlates with immune response to the human gut microbe Ruminococcus gnavus. Proc Natl Acad Sci USA 118:e2007595118. doi:10.1073/pnas.2007595118 33972416 PMC8157926

[31] Unverdorben LV, Pirani A, Gontjes K, Moricz B, Holmes CL, Snitkin ES, Bachman MA. 2025. Klebsiella pneumoniae evolution in the gut leads to spontaneous capsule loss and decreased virulence potential. mBio 16:e0236224. doi:10.1128/mbio.02362-24 40162782 PMC12077207

[32] Chen H, Liu H, Gong Y, Dunstan RA, Ma Z, Zhou C, Zhao D, Tang M, Lithgow T, Zhou T. 2024. A Klebsiella-phage cocktail to broaden the host range and delay bacteriophage resistance both in vitro and in vivo. NPJ Biofilms Microbiomes 10:127. doi:10.1038/s41522-024-00603-8 39543151 PMC11564825

[33] Zhao M, Li H, Gan D, Wang M, Deng H, Yang QE. 2024. Antibacterial effect of phage cocktails and phage-antibiotic synergy against pathogenic Klebsiella pneumoniae. mSystems 9:e0060724. doi:10.1128/msystems.00607-24 39166877 PMC11406915

[34] Yoo S, Lee K-M, Kim N, Vu TN, Abadie R, Yong D. 2024. Designing phage cocktails to combat the emergence of bacteriophage-resistant mutants in multidrug-resistant Klebsiella pneumoniae. Microbiol Spectr 12:e0125823. doi:10.1128/spectrum.01258-23 38018985 PMC10783003

[35] Qin J, Wu N, Bao J, Shi X, Ou H, Ye S, Zhao W, Wei Z, Cai J, Li L, Guo M, Weng J, Lu H, Tan D, Zhang J, Huang Q, Zhu Z, Shi Y, Hu C, Guo X, Zhu T. 2021. Heterogeneous Klebsiella pneumoniae co-infections complicate personalized bacteriophage therapy. Front Cell Infect Microbiol 10. doi:10.3389/fcimb.2020.608402 PMC7868542 33569355

[36] Mora-Quilis L, Sanjuán R, Domingo-Calap P. 2025. Reversible phenotypic resistance to phage infection via capsule downregulation in Klebsiella pneumoniae. iScience 28:114107. doi:10.1016/j.isci.2025.114107 41438077 PMC12719783

[37] Marchi J, Minh CNN, Debarbieux L, Weitz JS. 2025. Multi-strain phage induced clearance of bacterial infections. PLoS Comput Biol 21:e1012793. doi:10.1371/journal.pcbi.1012793 39903766 PMC11828373

[38] Zhu X, Xiao T, Jia X, Ni X, Zhang X, Fang Y, Hao Z. 2024. Isolation and evaluation of bacteriophage cocktail for the control of colistin-resistant Escherichia coli. Microb Pathog 197:107056. doi:10.1016/j.micpath.2024.107056 39442819

[39] Gu J, Liu X, Li Y, Han W, Lei L, Yang Y, Zhao H, Gao Y, Song J, Lu R, Sun C, Feng X. 2012. A method for generation phage cocktail with great therapeutic potential. PLoS One 7:e31698. doi:10.1371/journal.pone.0031698 22396736 PMC3291564

[40] Pyenson NC, Leeks A, Nweke O, Goldford JE, Schluter J, Turner PE, Foster KR, Sanchez A. 2024. Diverse phage communities are maintained stably on a clonal bacterial host. Science 386:1294–1300. doi:10.1126/science.adk1183 39666794 PMC7617280

[41] Ferriol-González C, Concha-Eloko R, Bernabéu-Gimeno M, Fernández-Cuenca F, Cañada-García JE, García-Cobos S, Sanjuán R, Domingo-Calap P. 2024. Targeted phage hunting to specific Klebsiella pneumoniae clinical isolates is an efficient antibiotic resistance and infection control strategy. Microbiol Spectr 12:e0025424. doi:10.1128/spectrum.00254-24 39194291 PMC11448410

[42] Wright RCT, Friman V-P, Smith MCM, Brockhurst MA. 2021. Functional diversity increases the efficacy of phage combinations. Microbiology (Reading, Engl) 167:001110. doi:10.1099/mic.0.001110 PMC8743627 34850676

[43] Yu P, Mathieu J, Li M, Dai Z, Alvarez PJJ. 2016. Isolation of polyvalent bacteriophages by sequential multiple-host approaches. Appl Environ Microbiol 82:808–815. doi:10.1128/AEM.02382-15 26590277 PMC4725286

[44] Castledine M, Buckling A. 2024. Critically evaluating the relative importance of phage in shaping microbial community composition. Trends Microbiol 32:957–969. doi:10.1016/j.tim.2024.02.014 38604881

[45] Moreno-Gámez S. 2022. How bacteria navigate varying environments. Science 378:845. doi:10.1126/science.adf4444 36423298

[46] Lee H, Popodi E, Tang H, Foster PL. 2012. Rate and molecular spectrum of spontaneous mutations in the bacterium Escherichia coli as determined by whole-genome sequencing. Proc Natl Acad Sci USA 109:E2774–E2783. doi:10.1073/pnas.1210309109 22991466 PMC3478608

[47] Schroeder JW, Yeesin P, Simmons LA, Wang JD. 2018. Sources of spontaneous mutagenesis in bacteria. Crit Rev Biochem Mol Biol 53:29–48. doi:10.1080/10409238.2017.1394262 29108429 PMC5975382

[48] Ozbudak EM, Thattai M, Kurtser I, Grossman AD, van Oudenaarden A. 2002. Regulation of noise in the expression of a single gene. Nat Genet 31:69–73. doi:10.1038/ng869 11967532

[49] Süel GM, Kulkarni RP, Dworkin J, Garcia-Ojalvo J, Elowitz MB. 2007. Tunability and noise dependence in differentiation dynamics. Science 315:1716–1719. doi:10.1126/science.1137455 17379809

[50] Yu Z, Luong T, Banuelos S, Sue A, Ryu H, Segal R, Roach DR, Huang Q. 2024. Leveraging mathematical modeling framework to guide regimen strategy for phage therapy. PLoS Complex Syst 1:e0000015. doi:10.1371/journal.pcsy.0000015

[51] Domingo-Calap P, Beamud B, Mora-Quilis L, González-Candelas F, Sanjuán R. 2020. Isolation and characterization of two Klebsiella pneumoniae phages encoding divergent depolymerases. Int J Mol Sci 21:3160. doi:10.3390/ijms21093160 32365770 PMC7246685

[52] Wingett SW, Andrews S. 2018. FastQ Screen: a tool for multi-genome mapping and quality control. F1000Res 7:1338. doi:10.12688/f1000research.15931.2 30254741 PMC6124377

[53] Prjibelski A, Antipov D, Meleshko D, Lapidus A, Korobeynikov A. 2020. Using SPAdes de novo assembler. Curr Protoc Bioinformatics 70:e102. doi:10.1002/cpbi.102 32559359

[54] Li H. 2013. Aligning sequence reads, clone sequences and assembly contigs with BWA-MEM. arXiv. 10.48550/arXiv.1303.3997.

[55] Bouras G, Nepal R, Houtak G, Psaltis AJ, Wormald P-J, Vreugde S. 2023. Pharokka: a fast scalable bacteriophage annotation tool. Bioinformatics 39:btac776. doi:10.1093/bioinformatics/btac776 36453861 PMC9805569

[56] Hackl T, Ankenbrand M, van AB, Wilkins D, Haslinger K. 2024. Gggenomes: effective and versatile visualizations for comparative genomics. arXiv. doi:10.48550/arXiv.2411.13556.

[57] Hockenberry AJ, Wilke CO. 2021. BACPHLIP: predicting bacteriophage lifestyle from conserved protein domains. PeerJ 9:e11396. doi:10.7717/peerj.11396 33996289 PMC8106911

[58] Moraru C, Varsani A, Kropinski AM. 2020. VIRIDIC-a novel tool to calculate the intergenomic similarities of prokaryote-infecting viruses. Viruses 12:1268. doi:10.3390/v12111268 33172115 PMC7694805

[59] Altschul SF, Gish W, Miller W, Myers EW, Lipman DJ. 1990. Basic local alignment search tool. J Mol Biol 215:403–410. doi:10.1016/S0022-2836(05)80360-2 2231712

[60] Concha-Eloko R, Stock M, De Baets B, Briers Y, Sanjuán R, Domingo-Calap P, Boeckaerts D. 2024. DepoScope: accurate phage depolymerase annotation and domain delineation using large language models. PLoS Comput Biol 20:e1011831. doi:10.1371/journal.pcbi.1011831 39102416 PMC11326577

[61] Vieira MF, Duarte J, Domingues R, Oliveira H, Dias O. 2025. PhageDPO: a machine-learning based computational framework for identifying phage depolymerases. Comput Biol Med 188:109836. doi:10.1016/j.compbiomed.2025.109836 39951981

[62] Magill DJ, Skvortsov TA. 2023. DePolymerase Predictor (DePP): a machine learning tool for the targeted identification of phage depolymerases. BMC Bioinformatics 24:208. doi:10.1186/s12859-023-05341-w 37208612 PMC10199479

