# Supplementary material for: Phenotypic heterogeneity shapes phage resistance and cocktail efficacy in Klebsiella pneumoniae
Source: Microbiol Spectr. 2026 Apr 30;14(6):e02261-25. doi: 10.1128/spectrum.02261-25 (PMC13228050; doi:10.1128/spectrum.02261-25)
Supplement: Fig. S1 — Genomic characterization of isolated phages. [file spectrum.02261-25-s0001.pdf]

Figure S1

A. Pairwise intergenomic similarity (VIRIDIC)

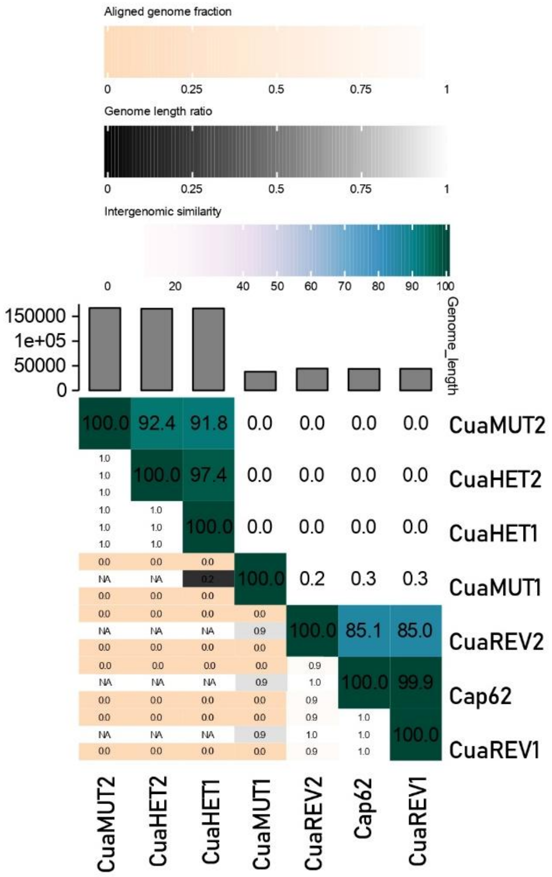

B. Predicted phage lifestyle (BACPHLIP)

Phage	Virulent	Temperate
**Cap62**	1.0	0.0
**CuaREV1**	1.0	0.0
**CuaREV2**	0.9752481267495525	0.024751873250447337
**CuaMUT1**	0.9981617952123443	0.001838204787655567
**CuaMUT2**	0.9745052770448549	0.02549472295514512
**CuaHET1**	0.9620052770448548	0.037994722955145124
**CuaHET2**	0.9620052770448548	0.037994722955145124

C. Depolymerase prediction

Phage	CDS	Name	DepoScope	PhageDPO	DePP
**Cap62**	007	Tail fiber protein	1.00	0.99	0.94
	056	Tail protein	1.00	0.91	0.98
	060	Tail fiber protein	1.00	1.00	0.96
**CuaREV1**	007	Tail fiber protein	1.00	0.99	0.95
	056	Tail protein	1.00	0.91	0.97
	060	Tail fiber protein	1.00	1.00	0.95
**CuaREV2**	007	Tail fiber protein	1.00	0.97	0.96
	056	Tail protein	1.00	0.93	0.96
	060	Tail fiber protein	1.00	0.95	0.97
**CuaMUT1**	046	Tail protein	1.00	0.97	0.96
**CuaMUT2**	nd	nd	-	-	-
**CuaHET1**	nd	nd	-	-	-
**CuaHET2**	nd	nd	-	-	-

Phage coding sequences with predicted score ≥ 0.90 by all three depolymerase predictors are shown.

nd: No coding sequences were predicted ass depolymeraes by all three predictors.

Figure S1. Genomic characterization of isolated phages. A. Intergenomic similarity matrix calculated by VIRIDIC. **B.** Phage lifestyle prediction assessed by BACPHLIP, indicating a virulent lifestyle for all isolates. **C.** *In silico* prediction of depolymerase activity using DepoScope, PhageDPO, and DePP.
